# Modulation of glucocorticoid receptor function under iron overload

**DOI:** 10.3389/fimmu.2025.1605420

**Published:** 2025-06-18

**Authors:** Wanting Zhu, Tineke Vanderhaeghen, Steven Timmermans, Jolien Vandewalle, Melanie Eggermont, Nicolette J. D. Verhoog, Claude Libert

**Affiliations:** ^1^ Center for Inflammation Research, Vlaams Instituut voor Biotechnologie (VIB), Ghent, Belgium; ^2^ Department of Biomedical Molecular Biology, Ghent University, Ghent, Belgium; ^3^ Department of Biochemistry, Stellenbosch University, Stellenbosch, South Africa

**Keywords:** iron, glucocorticoids, ferroptosis, function, critical illnesses

## Abstract

Acute iron overload leads to ferroptosis, in a mouse model of FeSO_4_ challenge causing lethal shock, associated with inflammation and multiple organ failure (MOF). We investigated molecular aspects causing this phenomenon upon FeSO_4_ overload, with a focus on the glucocorticoid receptor (GR), an important anti-inflammatory transcription factor. We report that Fe overload activates the HPA axis, leading to corticosterone increases in the blood, acutely causing upregulation of GR-dependent genes in liver. Using a GR blocker, mice with a reduced GR dimerization potential and removal of adrenal glands sensitizes mice for Fe-induced toxicity, GR appears essential to resist ferroptosis. However, stimulating GR with DEX is unable to protect mice against FeSO_4_-induced MOF and death. This dilemma is shown, by RNA sequencing, to be the result of a quick and complete inactivation of GR biological function by Fe^2+^, shortly after the initial activation. This inactivity of GR seems to be the result of a complete lack of GR to bind its ligand. We discuss the possible mechanism and complications for ferroptosis progression during diseases.

## 1 Introduction

Iron is an essential element for life, indispensable for various physiological processes, including oxygen transport, DNA synthesis, and electron transport (1, 2). Iron homeostasis is tightly regulated by a controlled feedback mechanism at both systemic and cellular levels. Excessive accumulation of its free form, non-transferrin-bound iron (NTBI), also known as catalytic iron, can be highly toxic, as it promotes the generation of toxic reactive oxygen species (ROS) through the Fenton and Haber-Weiss reaction. This process exacerbates oxidative stress, potentially leading to DNA damage and lipid peroxidation (3–5). This suggests that iron can act as a main inducer of a newly identified form of cell death called ferroptosis, characterized by excessive catalytic iron accumulation, lipid peroxidation, and eventually cell membrane rupture (4, 6). Next to its involvement in ferroptosis, iron is also an essential nutrient for bacterial proliferation. Therefore, excessive accumulation of iron could increase the risk of infections, which might be harmful. Altogether, iron overload can induce elevated oxidative stress, drive ferroptosis, facilitate bacterial proliferation, ultimately leading to cell death and tissue damage (5). These characteristics of iron and its homeostatic regulation are intrinsically linked to the physiological response to inflammation and infection. This is closely associated with the pathogenesis and progression of various inflammatory diseases, such as sepsis (7). Studies have shown that increased iron levels in the serum of sepsis patients positively correlate with both organ failure and mortality (8).

Glucocorticoids (GCs) are steroid hormones that are mainly synthesized and released by the adrenal cortex. Their secretion is regulated by the hypothalamic-pituitary-adrenal (HPA) axis and exhibits both a circadian and an ultradian pattern (9). Upon exposure to inflammation or a stress condition, the activity of the HPA axis is enhanced, thereby initiating the secretion of corticotropin-releasing hormone (CRH) by the hypothalamus. CRH will bind to its receptor CRHR1 in the anterior pituitary gland to promote the synthesis and secretion of adrenocorticotropic hormone (ACTH), which stimulates the adrenal glands to release GCs into the systemic circulation (10). Once in the bloodstream, GCs diffuse through cellular membranes and bind to the glucocorticoid receptor (GR) present in the cytoplasm. Without binding biologically active GCs, the GR remains in the cytoplasm in a multiprotein complex. Ligand binding induces a conformational change in the GR, leading to its dissociation from this complex of molecular chaperones, including heat shock proteins (e.g. HSP90 and HSP70). This conformational change also exposes its nuclear localization signal (NLS), promoting translocation of the GC-GR complex into the nucleus (11). Once inside the nucleus, GR can bind as a monomer or a homodimer to glucocorticoid response elements (GREs) in the promoter regions of its target genes, thereby modulating the transcription of a diverse number of genes involved in maintaining homeostasis, regulating metabolic pathways, and exerting potent anti-inflammatory and immunosuppressive effects (12–14). Additionally, GR can interact with other transcription factors, such as NF-κB and AP-1, through protein-protein interactions and repress the expression of pro-inflammatory genes, further mediating the anti-inflammatory response (15).

Activation of the HPA axis is essential for survival in various infectious diseases, including sepsis (16–18). However, in septic conditions, GC resistance develops, impairing the effectiveness of GCs in improving clinical outcomes (16). GC resistance is characterized by a diminished sensitivity or responsiveness to GCs, despite normal or elevated concentrations of these hormones (16, 19). Clinical and experimental evidence indicates a strong correlation between the degree of GC resistance, disease severity, and mortality in septic shock (20). The pathogenesis of GC resistance is complex and involves multiple mechanisms. Pro-inflammatory cytokines and oxidative stress are known to interfere with the GC signaling pathway at various stages (21). How GC resistance contributes to the progression of sepsis is not entirely clear, but the essential role of GR in hepatocytes to stimulate gluconeogenesis, which is involved in the removal of lactate, seems to be an essential function of GR that is lost in sepsis (16).

GR and iron exhibit a mutually regulatory relationship. Iron overload activates the HPA axis, leading to increased serum GC levels in rats (22). At the same time, GCs play a role in regulating iron metabolism. He et al. have shown that systemic GC administration increases the expression of iron regulatory protein-1 (IRP-1) and transferrin receptor-1 (TFR1), while downregulating ferritin in the liver, leading to increased liver iron accumulation in rats (23). Similarly, in an *in vitro* study of hippocampal neurons, exposure to GCs elevates intracellular iron levels and reduces ferritin expression, promoting oxidative damage of the neurons (24). Taken together, conditions such as sepsis which are characterized by iron accumulation (25) and GC resistance (16) suggest a potential mechanistic link between iron metabolism and GR function. Understanding this interaction may provide new insights into the pathophysiology of such diseases and may offer novel therapeutic targets.

In this study, applying a recently published *in vivo* mouse model of toxic Fe overload and ferroptosis (26), we demonstrate that acute iron overload activates the HPA axis, which triggers an inflammatory response and initiates the ferroptosis pathway. We describe that on one hand GC/GR biology reduces the toxic effects of iron overload, but on the other hand, that iron strongly undermines GR biology, as it interferes with the binding of ligands to the GR, thereby preventing GR nuclear translocation and inducing GC resistance.

## 2 Materials & methods

### 2.1 Mice

Female C57BL/6J mice as well as bilaterally adrenalectomized (ADX) C57BL/6J mice were purchased from Janvier (Le Genest-St. Isle, France). ADX mice were ordered at the age of 7 weeks and bilateral adrenalectomy was performed two weeks before delivery. *GR^fl/fl^* (generously provided by Dr. Jan Tuckermann, Ulm, Germany) were crossed with *Albumin Cre* transgenic mice, and the offspring was intercrossed to generate *GR^fl/fl^ Albumin Cre^Tg/+^* (GR^AlbKO^) mice, all in a C57BL/6J background. GR^dim/dim^ mice were generated by Reichardt et al. (1998) (27) and kept on an FVB/N background (provided by Dr. Jan Tuckermann, Ulm, Germany). Heterozygous GR^dim/wt^ mice were intercrossed to generate GR^wt/wt^ and GR^dim/dim^ homozygous mutant mice. All offspring were genotyped by PCR on genomic DNA isolated from toe biopsies. Mice were housed in a temperature-controlled, specific pathogen free (SPF) air-conditioned animal house with 14 and 10h light/dark cycles and received food and water *ad libitum.* The drinking water of ADX mice was supplemented with 0.9% NaCl. All mice were used at the age of 8 – 12 weeks, and all experiments were approved by the institutional ethics committee for animal welfare of the Faculty of Sciences, Ghent University, Belgium.

### 2.2 Reagents

Iron (II) sulfate heptahydrate, sodium sulfate anhydrous and LPS from *Salmonella abortusequi* were purchased from Sigma-Aldrich N.V. and dissolved in 0.9% NaCl. For intraperitoneal (i.p.) dexamethasone (DEX) injection, Rapidexon (Medini N.V.) was used. DEX was diluted in pyrogen-free phosphate buffered saline (PBS). RU486 (Mifepristone, Sigma-Aldrich N.V.), a GR antagonist, was diluted in DMSO. An overview of all reagents and tools used can be found in **Table 1**.

**Table 1:** Reagents and tools table.

Reagent or Resource	Source	Identifier
Experimental models: Organisms/strain		
C57BL/6J	Janvier	N/A
ADX C57BL/6J	Janvier	N/A
Nr3c1tm3Gsc/Nr3c1tm3Gsc Mus musculus (in manuscript referred to as GRdim/dim)	Jan Tuckermann (FVB background)	MGI Cat# 4455054 RRID: MGI:4455054
B6.Cg-Speer6-ps1Tg(Alb-cre)21Mgn/J Mus musculus (in manuscript referred to as Alb cre)	Dirk Elewaut	IMSR Cat# JAX:003574, RRID: IMSR_JAX:003574
Antibodies		
GR antibody (G-5)	Santa-Cruz Biotechnology	Cat# Sc-3932; RRID: AB_2687823
β-actin antibody (BA3R)	Thermo Fisher Scientific	Cat# MA5-15739; RRID: AB_10979409
Lamin A/C	Cell Signaling Technology	Cat# 2032, RRID: AB_2136278
β-tubulin antibody	Sigma-Aldrich N.V.	Cat# T4026, RRID: AB_477577
Amersham ECL anti-mouse antibody	GE Healthcare Life Sciences	Cat# NA931
Oligonucleotides		
qPCR primers (**Table 2**)	This MS	N/A
Chemicals, enzymes and other reagents		
LPS	Sigma Aldrich N.V.	Cat# L5886
Rapidexon 2 mg/ml (DEX)	Medini N.V.	NA
RU486	Sigma Aldrich N.V.	Cat# M-8046
Iron (II) sulfate heptahydrate	Sigma Aldrich N.V.	Cat# F7002-250G-D
1-methyl-2-phenylindole	Santa Cruz	Cat# sc-253936
1,1,3,3-tetramethoxypropane	Sigma Aldrich N.V.	Cat# 108383
[3H]-corticosterone	AEC Amersham	Cat# TRK 406
Sodium sulfate anhydrous	Sigma Aldrich N.V.	Cat# PHR2658
cOmplete™, EDTA-free Protease Inhibitor Cocktail	Sigma Aldrich N.V.	Cat# 11873580001
Software		
GraphPad Prism v.10	GraphPad Software	GraphPad Prism; RRID: SCR_002798
HOMER	(28)	HOMER; RRID: SCR_010881
IPA	Ingenuity Pathway Analysis (Qiagen)	IPA, RRID: SCR_008653
Other		
Aurum total RNA mini kit	Bio-Rad	Cat# 732-6820
Roche Light Cycler 480 instrument	Roche	RRID: SCR_020502
SensiFAST cDNA synthesis kit	GC Biotech BV	Cat# BIO-650504
ACTH ELISA kit	Abcam	Cat# Ab263880
Corticosterone ELISA kit	Arbor Assays	Cat# K014-H1; RRID: AB_2877626
SensiFAST SYBR No-ROX kit	GC Biotech BV	Cat# CSA-01190
Iron Assay kit	Abcam	Cat# Ab83366
Pierce™ BCA Protein Assay Kits	ThermoFisher Scientific	Cat# A65453
Sterilin™ Scintillation Vial	ThermoFisher Scientific	Cat# S31
IL-6 mouse ELISA kit	ThermoFisher Scientific	Cat# BMS603-2
Nuclear extract kit	Active Motif N.V.	Cat# 40010

### 2.3 Acute iron overload model

C57BL/6J mice treated with iron (II) sulfate heptahydrate received one i.p. injection of 300 mg/kg FeSO_4_·7H_2_O dissolved in 0.9% NaCl, with an injection volume of 200 μl per 20 g body weight. When pre-treated with DEX (10 mg/kg or 20 mg/kg), mice were also injected once i.p. with 300 mg/kg FeSO_4_. Using the GR inhibitor RU486, mice were first injected i.p. with RU486 (5 mg per mouse) or DMSO followed by a single i.p. injection of 150 mg/kg FeSO_4_. GR^dim/dim^, ADX, and GR^AlbKO^ mice were also injected once i.p. with 200 mg/kg FeSO_4_. For survival experiments, rectal body temperature was monitored and mice with a body temperature below 28°C were euthanized using cervical dislocation. For isolation experiments, mice were euthanized via cervical dislocation at the indicated time points and serum and organs were isolated. Blood was obtained via cardiac puncture after sedation with a ketamine/xylazine solution (Sigma-Aldrich N.V.). To obtain serum, blood samples were collected in 1.5 ml Eppendorf tubes, incubate overnight at 4°C and centrifuged at 10.000 rpm for 5 minutes at 4°C. Serum samples were stored at -20°C for biochemical analysis.

### 2.4 Endotoxemia experiment

C57BL/6J mice were injected i.p. with either PBS or DEX (10 mg/kg), followed by a lethal dose of LPS (22.5 mg/kg), dissolved in sterile PBS to induce endotoxin shock. For survival, rectal body temperature was monitored and mice with a body temperature below 28°C were euthanized *via* cervical dislocation.

### 2.5 Real-time quantitative PCR

Liver and kidney samples were isolated and stored in RNA later (Ambion, Life Technologies Europe) at -20°C until further processing. Total RNA was isolated using the Aurum total RNA mini kit (Biorad) according to manufacturer’s instructions. RNA concentration was measured with the Nanodrop 8000 (Thermo Fisher Scientific), and 1000 ng RNA was used to prepare cDNA with SensiFAST cDNA Synthesis Kit (Bioline). cDNA was diluted 20 times in ultrapure water for use in RT-qPCR reactions. RT-qPCR primers for used targets are listed in **Table 2**. RT-qPCR reaction was performed with SensiFAST SYBR No-ROX mix (Bioline) and was performed in duplicate in a Roche LightCycler480 system (Applied Biosystems). The stability of the housekeeping genes (HKGs) was determined by Genorm. Results are given as relative expression values normalized to the geometric mean of the house keeping genes *Rpl* and *Gapdh*, calculated using the 2^-ΔΔCt^method.

**Table 2:** Primer sequences used for RT-qPCR.

Gene	Forward primer (5′‐3′)	Reverse primer (5′‐3′)
*Rpl*	CCTGCTGCTCTCAAGGTT	TGGTTGTCACTGCCTCGTACTT
*Gapdh*	TGAAGCAGGCATCTGAGGG	CGAAGGTGGAAGAGTGGGAG
*Hmox1*	AAGCCGAGAATGCTGAGTTCA	GCCGTGTAGATATGGTACAAGGA
*Slc7a11*	GGCACCGTCATCGGATCAG	CTCCACAGGCAGACCAGAAAA
*Chac1*	CTGTGGATTTTCGGGTACGG	CCCCTATGGAAGGTGTCTCC
*Aimf2*	CTGCCTACCGCAGTGCATT	ACGCCATCATTTCTGCCCA
*Fth1*	CAAGTGCGCCAGAACTACCA	GCCACATCATCTCGGTCAAAA
*Slc39a14*	GTGTCTCACTGATTAACCTGGC	AGAGCAGCGTTCCAATGGAC
*Slc40a1*	ACCAAGGCAAGAGATCAAACC	AGACACTGCAAAGTGCCACAT
*Tfrc*	GTTTCTGCCAGCCCCTTATTAT	GCAAGGAAAGGATATGCAGCA
*Fam107a*	CAGACCAGAGTACAGAGAGTGG	GTGGTTCATAAGCAGCTCACG
*Fkbp5*	TGAGGGCACCAGTAACAATGG	CAACATCCCTTTGTAGTGGACAT
*Tsc22d3*	CCAGTGTGCTCCAGAAAGTGTAAG	AGAAGGCTCATTTGGCTCAATCTC
*Sgk1*	GAGATCGTGTTAGCTCCAAAGC	CTGTGATCAGGCATAGCACACT
*Tat*	TGCTGGATGTTCGCGTCAATA	CGGCTTCACCTTCATGTTGTC

### 2.6 Liver transcriptomics analysis

Female C57BL/6J mice were injected i.p. with 0.9% NaCl or 300 mg/kg FeSO_4_. After 6h, these mice were stimulated with PBS or DEX (10 mg/kg, i.p.) for 2h. Then, mice were euthanized, and livers were collected for RNA sequencing (RNAseq). Total RNA was isolated with Aurum total RNA mini kit (Biorad) according to the manufacturer’s instructions. RNA concentration was measured, and RNA quality was checked with the Agilent RNA 6000 Pico Kit (Agilent Technologies). The sequencing library was constructed using the Illumina TruSeq Stranded mRNA Library Prep kit according to the manufacturer’s instructions. The library was sequenced single-end for 100 cycles on an Illumina NovaSeq 6000 sequencing device. Reads were quality checked with FastQC (28) and mapped to the mm39 reference genome using GENCODE known splice junctions (v28) with the STAR (v2.7.10b) (29) with added option of “–quantMode GeneCounts” to directly obtain the counts per gene and differential expressed genes (DEGs) were found by the DESeq2 R package (30) with the false discovery rate (FDR) set at 5%. Motif finding multiple motifs or *de novo* motif finding was performed using the HOMER software. We used the promoter region (start offset: −1 kb, end offset: 50 bp downstream of transcription start site (TSS)) to search for known motif enrichment and *de novo* motifs (31). Visualizations were made using R software. Ingenuity Pathway Analysis (IPA, Qiagen) was utilized for the analysis of RNAseq data.

### 2.7 Biochemical analysis

Analysis of mouse plasma aspartate aminotransferase (AST), alanine aminotransferase (ALT), creatinine, urea, creatine kinase (CK) and lactate dehydrogenase (LDH) levels were kindly provided to us by the University Hospital of Ghent. ACTH (Abcam) and corticosterone (Arbor Assays) levels were measured in mouse serum with the use of colorimetric assays according to manufacturer’s instructions. Serum interleukin-6 (IL-6, eBioscience) levels were measured by ELISA.

### 2.8 Iron assay kit

Total iron, ferrous (Fe^2+^) and ferric (Fe^3+^) iron levels were measured in serum and liver samples using the Iron Assay Kit (Abcam) according to the manufacturers’ instructions. Briefly, serum samples were incubated at 37°C for 30 minutes. To measure total iron and Fe^3+^ levels, an iron reducer was added. Then, the Iron Probe was added and incubated at 37°C for 60 minutes. Samples were measured using a microplate reader at 595 nm. For liver samples, the samples were first homogenized in lysis buffer provided by the manufacturer.

### 2.9 Colorimetric lipid peroxidation assay

Malondialdehyde (MDA) levels were determined via a colorimetric assay in which 1-methyl-2-phenylindole species were measured, as previously described (26, 32). In short, serum samples were diluted in PBS to a final volume of 100 μl and then mixed with 325 μl of 1-methyl-2-phenylindole (Santa Cruz) dissolved in a mixture of acetonitrile/methanol (3:1), with 10 mM 1-methyl-2-phenylindole as a final concentration. Next, 75 µl of 37% hydrochloric acid was added and incubated at 70°C for 45 minutes. Then, the samples were centrifugated (10 minutes, 14.000 rpm, 4°C) and the supernatant was collected. The absorbance was measured at 595 nm, and a standard of 1,1,3,3-tetramethoxypropane (Sigma-Aldrich N.V.) was used as a source of MDA to determine its concentration.

### 2.10 Western blot analysis

For the detection of GR protein levels, total protein was isolated from snap frozen liver with RIPA lysis buffer, supplemented with protease inhibitor cocktail (Roche). Protein samples containing 20 µg of protein were separated by electrophoresis on an 8% gradient SDS-polyacrylamide gel and transferred to nitrocellulose filters (pore size 0,45 µm). After blocking the membranes with a ½ dilution of Starting Block/PBST 0.1% (Thermo Fisher Scientific), membranes were incubated overnight at 4°C with primary antibodies against GRα/β (1:1.000, G5, Santa Cruz), and β-actin (1:5.000, Life Technologies Europe) as an internal control. Blots were washed with 0.1% PBST and then incubated for 1h at room temperature with Amersham ECL anti-mouse antibody (1:2.000, GE Healthcare Life Sciences). Immunoreactive bands were visualized and quantified using an Amersham Imager 600 (GE Healthcare Life Sciences).

### 2.11 Nuclear translocation

GR nuclear translocation was assayed in liver samples derived from C57BL/6J mice that were injected with 10 mg/kg DEX or PBS 6h after 300 mg/kg FeSO_4_ or 0.9% NaCl challenge. Nuclear proteins were isolated out of freshly isolated liver tissue using the nuclear extract kit according to manufacturer’s instructions (Active Motive). Western blot analysis was carried out as described above. Lamin A/C (1:100, Cell Signaling Technology) and β-tubulin (1:1.000, Sigma-Aldrich N.V.) was used as an internal control for nuclear and cytoplasmic extract respectively.

### 2.12 Liver cytosolic GR ligand binding

The whole livers from ADX mice were thawed, cut into fine pieces, and homogenized with a Miccra D-1 homogenizer in 1.5 ml cytosol buffer (10 mM Tris _HCl, pH 7.5, containing 0.25 M sucrose, 0.1 mM PMSF, and protease inhibitor tablets (Roche)) on ice. Each homogenate was ultracentrifuged at 100.000 g for 1h at 4°C. The upper fatty layer was discarded, and the crude cytosol was treated with 100 µl dextran-coated charcoal (DCC; 3.75 g of charcoal and 0.375 g of dextran T-70 made up in 100 ml of 10 mM Tris HCl, pH 7.5) for 15 minutes on ice to remove endogenous steroids. Each sample was then centrifuged at 3.000 g for 15 minutes at 4°C. The clear supernatant was referred to as “liver cytosol” and diluted to a final volume of 2 ml. The protein concentration of the liver cytosol was determined using the bicinchoninic acid assay (Pierce).

To determine the GR binding capacity of the liver cytosols, 250 µl liver cytosol was incubated with 10 µl 400 nM [3H]-corticosterone (specific activity of 79 Ci/mmol (AEC Amersham)) in the presence of 10 µl ethanol (total binding) or 10 µl excess (0.4 mM) unlabeled corticosterone (non-specific binding) and 130 µl cytosol buffer for 24h at 4°C. Next, 100 µl DCC was added to each sample and incubated on ice for 10 minutes to separate free radiolabeled ligand from bound, followed by centrifugation at 3.000 g for 10 minutes at 4°C. The supernatants (300 µl) were transferred into scintillation vials containing 2.5 ml scintillation fluid (Thermo Scientific), vortexed and the counts per minute were measured using a Tri-Carb 2810TR scintillation counter (PerkinElmer). Specific binding was determined as the difference between total binding and non-specific binding and was normalized for protein content (expressed as fmol/mg protein). DCC efficiency for removing unbound radioactive-labelled steroids was at least 90%. The counting efficiency was 44%, and ligand depletion was below 10%.

### 2.13 Statistics

Data were expressed as means ± standard errors of the means (SEM). Statistical significance was evaluated with two-way unpaired Student’s t-test and two-way ANOVA in GraphPad Prism 10.0 software (GraphPad Software, San Diego, CA). If applicable, two-way ANOVA analyses were followed by *post-hoc* analysis to correct for multiple testing during the pairwise multiple comparisons using the Šídák’s multiple comparisons test. Fold changes or ratios were log(Y) transformed before statistical analysis. Survival curves were subjected to the Log-Rank (Mantel-Cox) test to investigate whether statistical significance could be observed during different groups. A P-value of < 0.05 was considered statistically significant. ****P ≤ 0.0001, ***P ≤ 0.001, **P ≤ 0.01, *P ≤ 0.05, ns = not significant.

## 3 Results

### 3.1 Genome-wide overview of FeSO_4_-induced transcriptional changes in the liver

**Figure 1:**
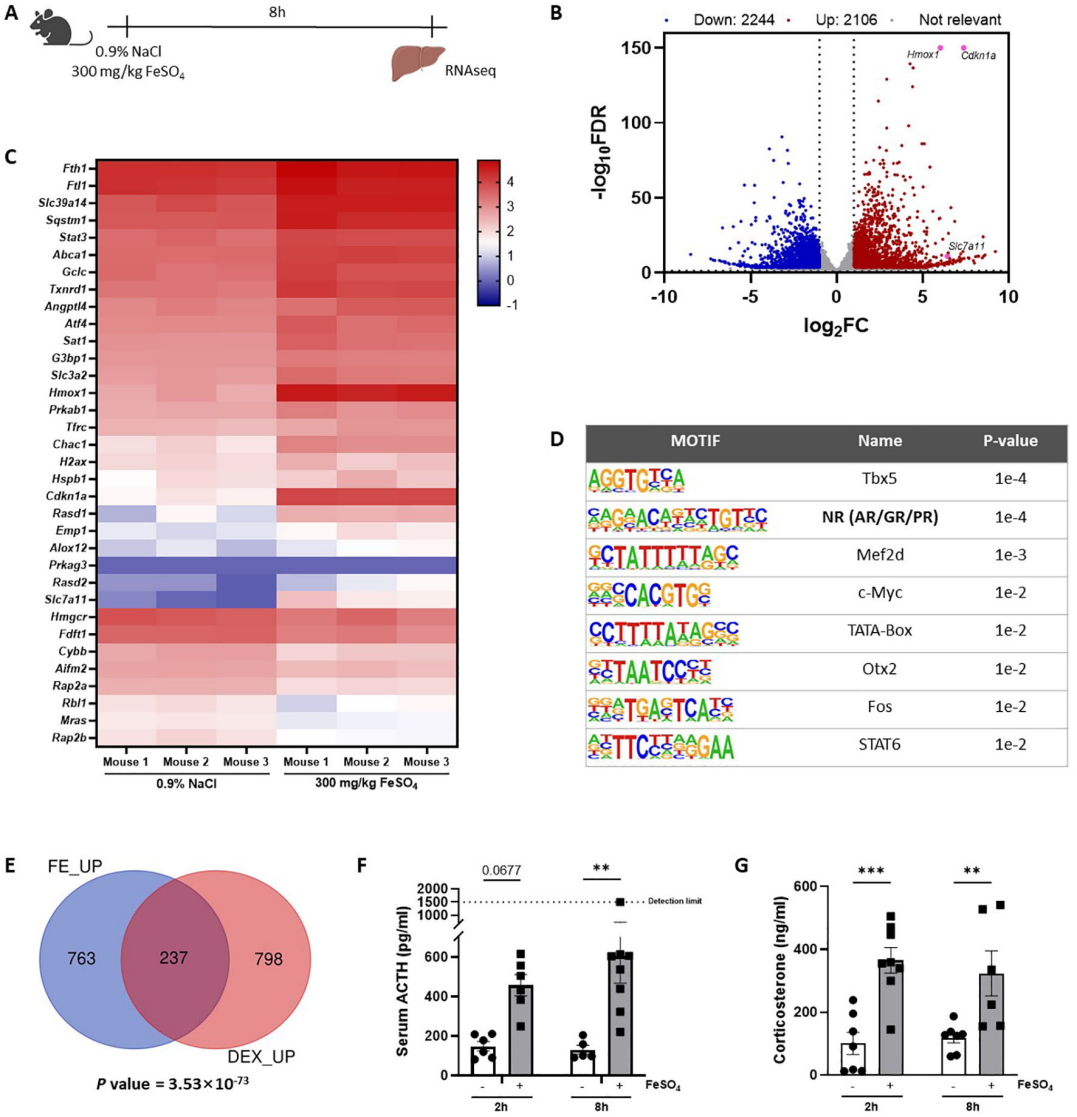
Genome-wide overview of FeSO_4_-induced transcriptional changes in the liver. **(A)** Experimental set-up: C57BL/6J mice were injected with 300 mg/kg FeSO_4_ or 0.9% NaCl intraperitoneally (i.p.). The liver was isolated after 8h for RNAseq (n = 3/group). **(B)** Volcano plot of genes upregulated and downregulated by 300 mg/kg FeSO_4_ (FDR ≤ 0.05, |LFC| > 1). Upregulated genes are indicated in red, downregulated genes are indicated in blue. **(C)** Heatmap represents log_10_ values of differentially expressed genes (counts) involved in the ferroptosis signaling pathway identified *via* IPA per mouse included in the 0.9% NaCl group and the FeSO_4_ group. **(D)** HOMER motif analysis of top 1000 genes upregulated by 300 mg/kg FeSO_4_ (start offset: -1 kb, end offset: 50 bp downstream of TSS). **(E)** C57BL/6J mice were stimulated i.p. with 300 mg/kg FeSO_4_ for 8h or 10 mg/kg DEX for 2h. Mouse livers were isolated at the indicated timepoints and the effect of FeSO_4_ and DEX stimulation was studied *via* RNAseq (n=3 per group). Venn diagram depicting the overlap of the top 1.000 upregulated genes by FeSO_4_ (FDR ≤ 0.05) or the genes induced by DEX (FDR ≤ 0.05, LFC > 0). A hypergeometric test was used to obtain a p-value of the overlap. **(F, G)** C57BL/6J mice were injected with 300 mg/kg FeSO_4_ or 0.9% NaCl i.p. Serum was collected after 2h and 8h. ACTH **(F)** and corticosterone levels **(G)** were measured. N = 5-8 per group, two independent experiments. Data information: All bars represent mean ± SEM. P-values were calculated using two-way ANOVA followed by *post-hoc* Šídák’s multiple comparisons test to correct for multiple testing during the pairwise multiple comparisons, except if otherwise stated. ***P ≤ 0.001, **P ≤ 0.01.

To study the *in vivo* effects of iron overload, an experimental mouse model was applied, previously described by Van Coillie et al. (26). A dose of 300 mg/kg FeSO_4_ was required to induce lethal shock (**Supplementary Figures 1A, B**), while equal amounts of Na_2_SO_4_ and NaCl remained safe. To study molecular mechanisms of Fe overload, and since the liver plays a central role in iron metabolism (33), mice were injected with either 0.9% NaCl or 300 mg/kg FeSO_4_, the livers were isolated 8h later and a genome-wide analysis was performed *via* RNAseq (**Figure 1A**). When plotting the log fold changes (LFCs) of all FeSO_4_ responsive genes (|LFC| > 1, FDR ≤ 0.05), compared to the NaCl group, 2.106 genes were upregulated by FeSO_4_, including *Cdkn1a, Hmox1*, and *Slc7a11* (**Figure 1B**), genes known to be involved in ferroptosis (6, 34). Additionally, 2.244 genes were downregulated. After excluding genes encoding proteins without known function (all Rik genes and Gm genes e.g. A330009N23Rik and Gm10030), IPA of DEGs (FDR ≤ 0.05, |LFC| > 1, |Z score| ≥ 1) identified the activation of inflammation-related pathways, such as IL-6 signaling, as well as ferroptosis in response to acute iron overload (**Table 3**). As expected, serum IL-6 levels were significantly increased 8h after FeSO_4_ challenge (300 mg/kg) compared to their controls (**Supplementary Figure 1C**). Furthermore, the expression level of genes associated with the ferroptosis pathway identified *via* IPA are shown in **Figure 1C**. Ferroptosis-related genes including *Slc7a11*, *Chac1* and *Hmox1* were significantly upregulated. Additionally, the expression of genes involved in iron import (*Slc39a14, Tfrc*) and iron storage (*Fth1, Ftl1*) were also significantly increased. Conversely, the anti-ferroptosis gene (35) *Fsp1* (officially renamed as *Aifm2*) was downregulated (**Figure 1C**).

**Table 3:** IPA of differentially expressed genes after acute iron overload.

Pathway	-log(P-value)	Z-score
1. Role of Osteoclasts in Rheumatoid Arthritis Signaling Pathway	4.63	1.195
2. Cardiac Hypertrophy Signaling (Enhanced)	4.59	2.64
3. NFE2L2 regulating anti-oxidant/detoxification enzymes	4.38	2.53
4. Interleukin-4 and Interleukin-13 signaling	4.32	3.087
5. Toll-like Receptor Signaling	4.18	1.414
6. Tumor Microenvironment Pathway	4.09	1.443
7. IL-6 Signaling	4.01	2.197
8. p38 MAPK Signaling	3.99	3.651
9. Sertoli Cell-Sertoli Cell Junction Signaling	3.94	2.433
10. Role of JAK family kinases in IL-6-type Cytokine Signaling	3.75	2.746
11. NGF-stimulated Transcription	3.66	2.84
12. TNFR2 Signaling	3.62	1.941
13. Hepatic Fibrosis Signaling Pathway	3.58	1.941
14. Cachexia Signaling Pathway	3.53	2.086
15. IL-1 Signaling	3.45	2.5
16. ERK5 Signaling	3.40	2.4
17. NRF2-mediated Oxidative Stress Response	3.39	1.671
18. Ceramide Signaling	3.33	1.225
19. Hepatic Cholestasis	3.31	2.335
20. HMGB1 Signaling	3.20	1.826
21. IL-17 Signaling	3.08	2.343
22. TNFR1 Signaling	2.75	1
23. iNOS Signaling	2.74	1.604
24. Cholecystokinin/Gastrin-mediated Signaling	2.70	1.347
**25. Ferroptosis Signaling Pathway**	**2.65**	**1.029**
26. CD27 Signaling in Lymphocytes	2.57	1.291
27. Salvage Pathways of Pyrimidine Ribonucleotides	2.55	1
28. Signaling by PTK6	2.55	1.213
29. Apoptosis Signaling	2.43	1.633
30. IL-17A Signaling in Fibroblasts	2.42	2.711

Pathway analysis was performed on all differentially expressed genes using IPA identified in the liver of mice injected with 300 mg/kg FeSO_4_ (FDR ≤ 0.05, |LFC| > 1, |Z score| ≥ 1). The top 30 of the functional enrichment results are depicted.The Ferroptosis Signaling Pathway is indicated in bold.

Next, a HOMER motif enrichment analysis was performed on the top 1.000 upregulated genes after excluding genes encoding for proteins without known function (**Figure 1D**). The promoter regions of these genes were significantly enriched for motifs associated with steroid receptors (36), which are commonly linked to the GR, androgen receptor (AR), and progesterone receptor (PR). Previous studies have demonstrated that iron can influence oxidative stress and inflammation (7, 37), potentially interfering with the HPA axis. This prompted us to explore the role of GR in the acute iron overload model. To identify whether FeSO_4_ is able to induce the expression of GR responsive genes, bulk RNAseq was performed on livers isolated from mice stimulated with DEX (10 mg/kg) for 2h, a strict GR agonist, or PBS as a control. The overlap between DEX-induced genes (FDR ≤ 0.05, LFC > 0) and the top 1.000 upregulated genes by FeSO_4_ demonstrate a significant (P = 3.53×10^-73^) enrichment of GR responsive genes in the livers of mice challenged with FeSO_4_ (**Figure 1E**), indicating that iron activates the GR. Since it is very unlikely that iron directly activates GR, we studied if iron stimulates the HPA axis. Indeed, serum ACTH (**Figure 1F**) and corticosterone (**Figure 1G**) levels significantly increased 2h and 8h after FeSO_4_ injection, and the expression of typical GR-responsive genes measured *via* RT-qPCR were significantly induced in the liver by FeSO_4_. This induction was absent in ADX mice lacking endogenous GC production (**Supplementary Figure 1D**), which proves that FeSO_4_ activates GR, and thus GRE genes, through the activation of the HPA axis and the production of GCs.

Taking together, these findings suggest that a lethal dose of iron has widespread effects on the liver, notably activating GR signaling, inflammation, and the ferroptosis pathway. This highlights the role of iron in modulating stress-related biological processes and inducing cell death mechanisms, such as ferroptosis.

### 3.2 Dexamethasone is unable to protect against FeSO_4_-induced lethal shock

**Figure 2:**
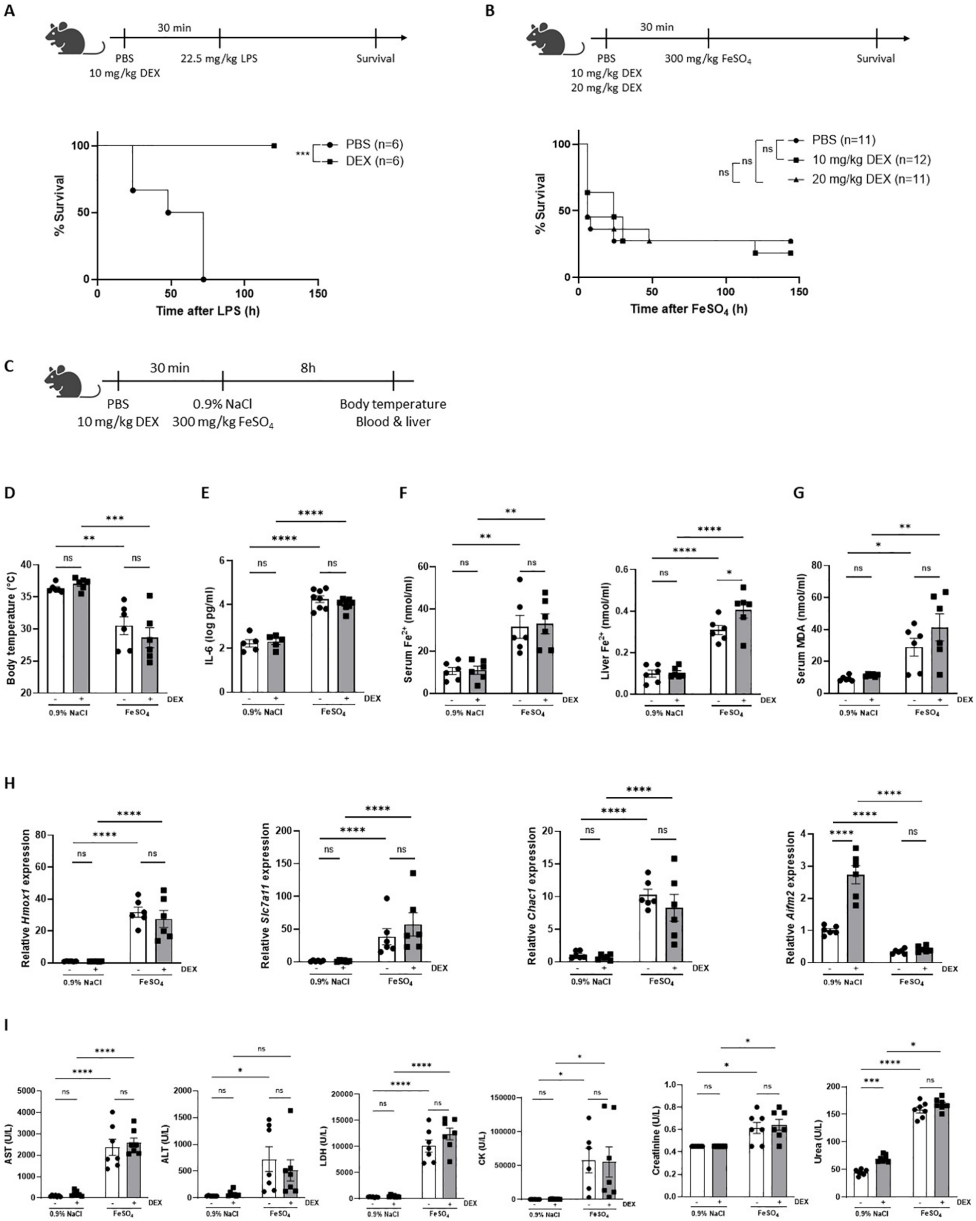
Dexamethasone is unable to protect against FeSO_4_-induced lethal shock. **(A)** C57BL/6J mice were pretreated with 10 mg/kg DEX or PBS 30 minutes before injection of 22.5 mg/kg LPS. Survival was monitored. N-values are indicated in the figure legend. **(B)** C57BL/6J mice were pretreated with 10 mg/kg or 20 mg/kg DEX 30 minutes before injection of 300 mg/kg FeSO_4_. Survival was monitored. N-values are indicated in the figure legend. **(C)** Experimental setup: C57BL/6J mice were pretreated with 10 mg/kg DEX or vehicle (PBS) for 30 minutes, followed by injection of 0.9% NaCl or 300 mg/kg FeSO_4_. After 8h, body temperature was measured, and serum and livers were isolated for analysis. **(D)** Body temperature, n = 6 per group, two independent experiments. **(E)** Serum IL-6 levels, n = 5-8 per group, two independent experiments. **(F, G)** Serum Fe^2+^, liver Fe^2+^ concentration **(F)** and serum MDA levels **(G)**, n=6 per group, two independent experiments. **(H)** RT-qPCR analysis of ferroptosis-related genes in liver. N = 6 per group, two independent experiments. **(I)** Serum AST, ALT, LDH, CK, creatinine, and urea levels, n= 6-8 per group, two independent experiments. Data information: All bars represent mean ± SEM. P-values were calculated using two-way ANOVA followed by *post-hoc* Šídák’s multiple comparisons test to correct for multiple testing during the pairwise multiple comparisons, except if otherwise stated. Survival curves were analyzed with a Log-Rank (Mantel-Cox) test. ****P ≤ 0.0001; ***P ≤ 0.001; **P ≤ 0.01; *P ≤ 0.05; ns, not significant.

Since DEX is characterized by its anti-inflammatory capacity and is known to protect against LPS-induced endotoxemia, a well-known acute lethal inflammatory model (38–40), we have included an LPS survival experiment with DEX pretreatment as proof-of-concept (**Figure 2A**). Since FeSO_4_ strongly activates GR in the liver (**Figure 1**), we investigated whether DEX is able to protect against a lethal dose of FeSO_4_ (300 mg/kg) using the same experimental set-up as in LPS-induced endotoxemia. C57BL/6J mice were treated with DEX (10 mg/kg or 20 mg/kg) or PBS followed by an i.p. injection of 300 mg/kg FeSO_4_ after 30 minutes. In contrast to a similar DEX treatment in LPS-induced endotoxemia, the DEX doses tested were unable to protect against a lethal dose of FeSO_4_ (**Figure 2B**). Acute iron overload can lead to multiple organ dysfunction driven by ferroptosis, which is characterized by elevated levels of catalytic iron and MDA, and serum injury markers (26). To investigate the effect of DEX, mice were pretreated with PBS or DEX 30 minutes before FeSO_4_ or 0.9% NaCl injection. After 8h, the body temperature was measured, serum was collected, and livers were isolated (**Figure 2C**). Acute iron overload caused severe hypothermia and DEX pretreatment failed to improve this (**Figure 2D**). Furthermore, serum IL-6 levels were significantly increased upon 300 mg/kg FeSO_4_ and DEX was not able to reduce these IL-6 levels measured in the serum (**Figure 2E**). As expected, mice injected with FeSO_4_ were characterized by a ferroptosis signature including accumulation of Fe^2+^, Fe^3+^ and total iron in serum and liver (**Figure 2F**, **Supplementary Figures 2A, B**), along with elevated serum MDA levels (**Figure 2G**). However, DEX pretreatment was unable to attenuate this ferroptosis signature. Furthermore, the expression of typical ferroptosis-related genes *Slc7a11*, *Chac1* and *Hmox1* were significantly upregulated in the liver after FeSO_4_ compared to 0.9% NaCl, while a gene repressed by ferroptosis (*Aifm2*) was significantly downregulated. Also here, DEX did not affect the expression of these genes (**Figure 2H**). Finally, serum injury markers including LDH, AST, ALT, creatinine, urea, and CK were measured. DEX is able to increase the expression of the carbamoylphosphate synthetase-I (CPS) gene (41), which forms the rate-limiting factor of the urea cycle. Furthermore, DEX can induce the transcription of argininosuccinate synthetase and lyase mRNA, and stabilize arginase mRNA and the protein ornithine transcarbamylase, enzymes involved in the urea cycle (42). As expected, urea was significantly increased in the serum of mice injected with 0.9% NaCl and pretreated with DEX. Furthermore, all serum injury markers were significantly elevated following FeSO_4_ administration, reflecting necrosis, and liver, kidney, and muscle damage. Pretreatment with DEX had no impact on these serum organ damage markers (**Figure 2I**). These data show that a DEX dose, able to prevent acute endotoxemia, has no protective effects on ferroptosis-associated multi-organ dysfunction.

### 3.3 FeSO_4_-induced lethal shock is controlled by GC/GR biology

**Figure 3:**
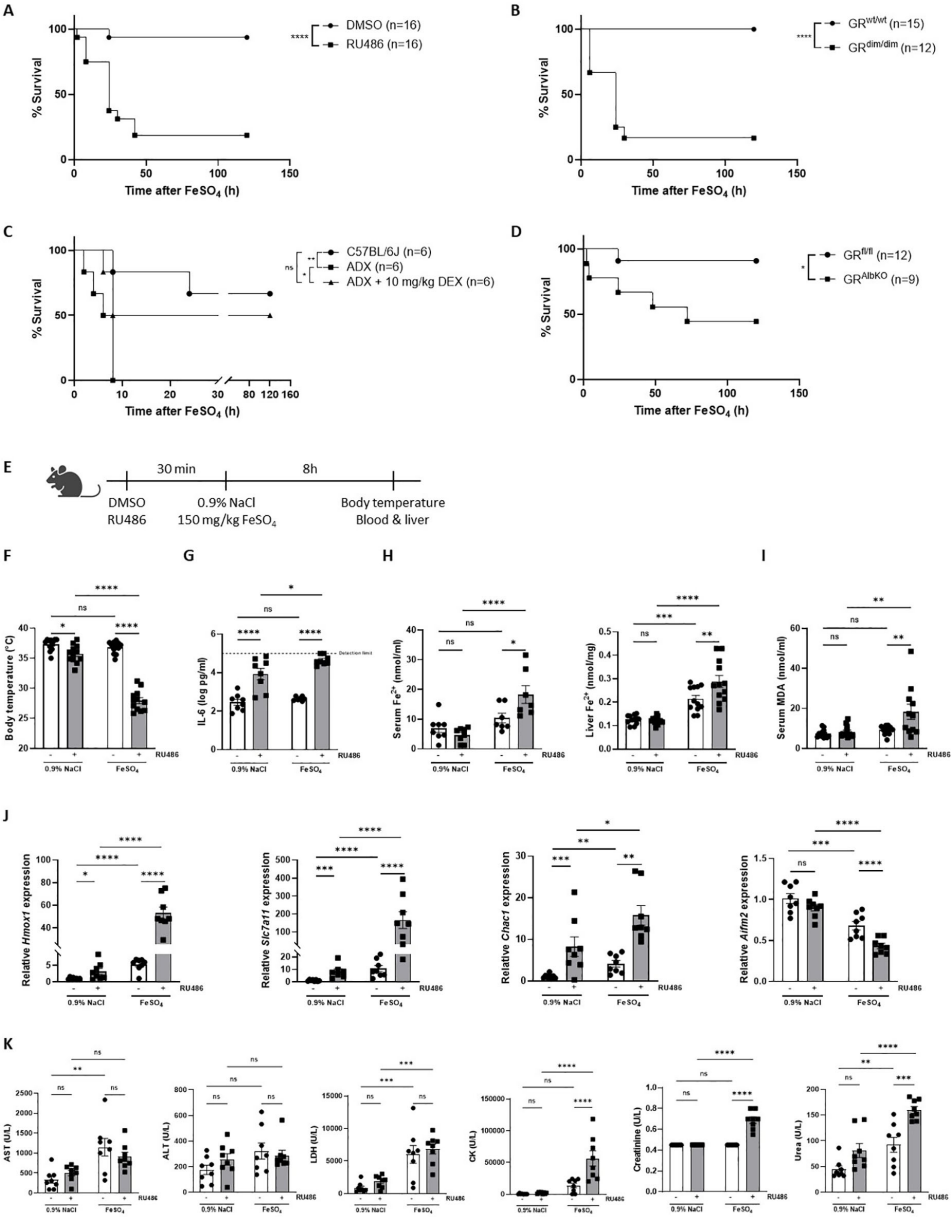
FeSO_4_-induced lethal shock is controlled by GC/GR biology. **(A)** C57BL/6J mice were pretreated with 5 mg RU486 or vehicle (DMSO) 30 minutes before injection of 150 mg/kg FeSO_4_. Survival was monitored for 5 days. N-values are indicated in the figure. **(B)** GR^dim/dim^ and wild-type littermates were injected with 200 mg/kg FeSO_4_, and survival was monitored for 5 days. N-values are indicated in the figure. **(C)** ADX and C57BL/6J mice were pretreated with 10 mg/kg DEX or PBS (vehicle) 30 minutes before an FeSO_4_ challenge (200 mg/kg). Survival was monitored for 5 days, n-values are depicted in the figure. **(D)** GR^AlbKO^ and GR^fl/fl^ mice were injected with 200 mg/kg FeSO_4_, and survival was monitored for 5 days. N-values are shown in the survival curve. **(E)** Experimental setup: C57BL/6J mice were pretreated with 5 mg RU486 or vehicle (DMSO) for 30 minutes, followed by injection of 0.9% NaCl or 150 mg/kg FeSO_4_. After 8h, body temperature was measured, and serum and livers were collected for analysis. **(F)** Body temperature, n = 8 per group, two independent experiments. **(G)** Serum IL-6 levels, n = 8 per group, two independent experiments. **(H, I)** Serum Fe^2+^, liver Fe^2+^ concentration **(H)** and serum MDA levels **(I)**, n= 7-12 per group, two independent experiments. **(J)** RT-qPCR analysis of ferroptosis-related genes in liver, n = 8 per group, two independent experiments. **(K)** Serum levels of AST, ALT, LDH, CK, creatinine, and urea, n = 7-8 per group, two independent experiments. Data information: All bars represent mean ± SEM. P-values were calculated using two-way ANOVA followed by *post-hoc* Šídák’s multiple comparisons test to correct for multiple testing during the pairwise multiple comparisons, except if otherwise stated. Survival curves were analyzed with a Log-Rank (Mantel-Cox) test. ****P ≤ 0.0001; ***P ≤ 0.001; **P ≤ 0.01; *P ≤ 0.05; ns, not significant.

Despite that DEX has no protective effects against any effect of iron overload phenomena including lethality, the fact that iron activates GR signaling might still demonstrate an involvement of GC/GR biology in iron metabolism in the liver (23). We decided to investigate the role of GR signaling under acute iron overload, in which a sublethal dose of FeSO_4_ was used. First, mice were pretreated with RU486, a GR antagonist (43), and challenged with FeSO_4_. RU486-pretreated mice showed a significantly higher mortality compared to the DMSO-treated control group (**Figure 3A**). Similarly, GR^dim/dim^ mice, which have an impaired GR dimerization potential (44), exhibited increased mortality compared to their wild-type littermates (**Figure 3B**). Furthermore, ADX mice also displayed a higher sensitivity to FeSO_4_, which could be reversed by pre-treatment with DEX (**Figure 3C**). Given the central role of liver in iron metabolism, the response to FeSO_4_-induced lethal shock was also determined in hepatocyte-specific GR knockout (GR^AlbKO^) mice. The absence of GR in hepatocytes also led to a higher mortality rate when these mice were challenged with FeSO_4_ compared to their wild-type littermates (**Figure 3D**), reinforcing the importance of GR signaling in liver-mediated protection against acute iron overload. Collectively, these findings underscore the critical role of proper GR signaling in the resistance to acute iron-induced lethal shock.

To further explore the mechanism behind the importance of GR in ferroptosis, mice were pretreated with RU486 or DMSO, 30 minutes before injection of a sublethal dose of FeSO_4_ or 0.9% NaCl. Parameters were measured 8h later (**Figure 3E**). A significant drop in body temperature was detected when mice were injected with RU486 and challenged with 150 mg/kg FeSO_4_ (**Figure 3F**). As GR is known as an anti-inflammatory protein (45), IL-6 levels were significantly increased in the serum of mice when GR was inhibited using RU486, as expected. Furthermore, although IL-6 levels were not increased in the serum of mice challenged with 150 mg/kg FeSO_4_, serum IL-6 levels were significantly higher when GR was inhibited combined with a low dose of FeSO_4_ (**Figure 3G**), indicating that blocking GR sensitizes mice to acute iron overload in terms of IL-6 levels. Fe^2+^ and Fe^3+^ iron levels were slightly increased after FeSO_4_ injection, however RU486 pretreatment led to a significant accumulation of iron in both the serum and liver (**Figure 3H**, **Supplementary Figure 3A**). Next, the expression levels of iron import genes (*Tfrc, Slc39a14*), iron storage genes (*Fth1*), and iron export genes (*Slc40a1*) were measured in the liver of these mice. Consistent with the iron accumulation in the liver, the expression of iron import and storage genes was significantly higher while genes involved in iron export showed a trend towards downregulation in RU486-pretreated mice following FeSO_4_ (**Supplementary Figure 3B**). Furthermore, serum MDA levels, used as an indicator of lipid peroxidation, were significantly higher in RU486-pretreated mice compared to their control groups after FeSO_4_ injection (**Figure 3I**). Additionally, the expression of ferroptosis related genes were significantly altered in the liver of these mice. The expression of *Slc7a11*, *Chac1*, as well as *Hmox1* were upregulated, while the ferroptosis suppressor *Aifm2* was downregulated in mice pretreated with RU486 and challenged with FeSO_4_ (**Figure 3J**). Furthermore, several serum injury markers, including CK, creatinine, and urea, were significantly elevated in the RU486-pretreated group compared to their control groups after FeSO_4_ (**Figure 3K**). These data indicate that disruption of GR signaling impairs iron metabolism, leading to an excessive iron accumulation and to the activation of ferroptosis signaling pathways, ultimately contributing to the increased lethality observed under conditions of acute iron overload.

### 3.4 FeSO_4_-induces genome-wide GC resistance

**Figure 4:**
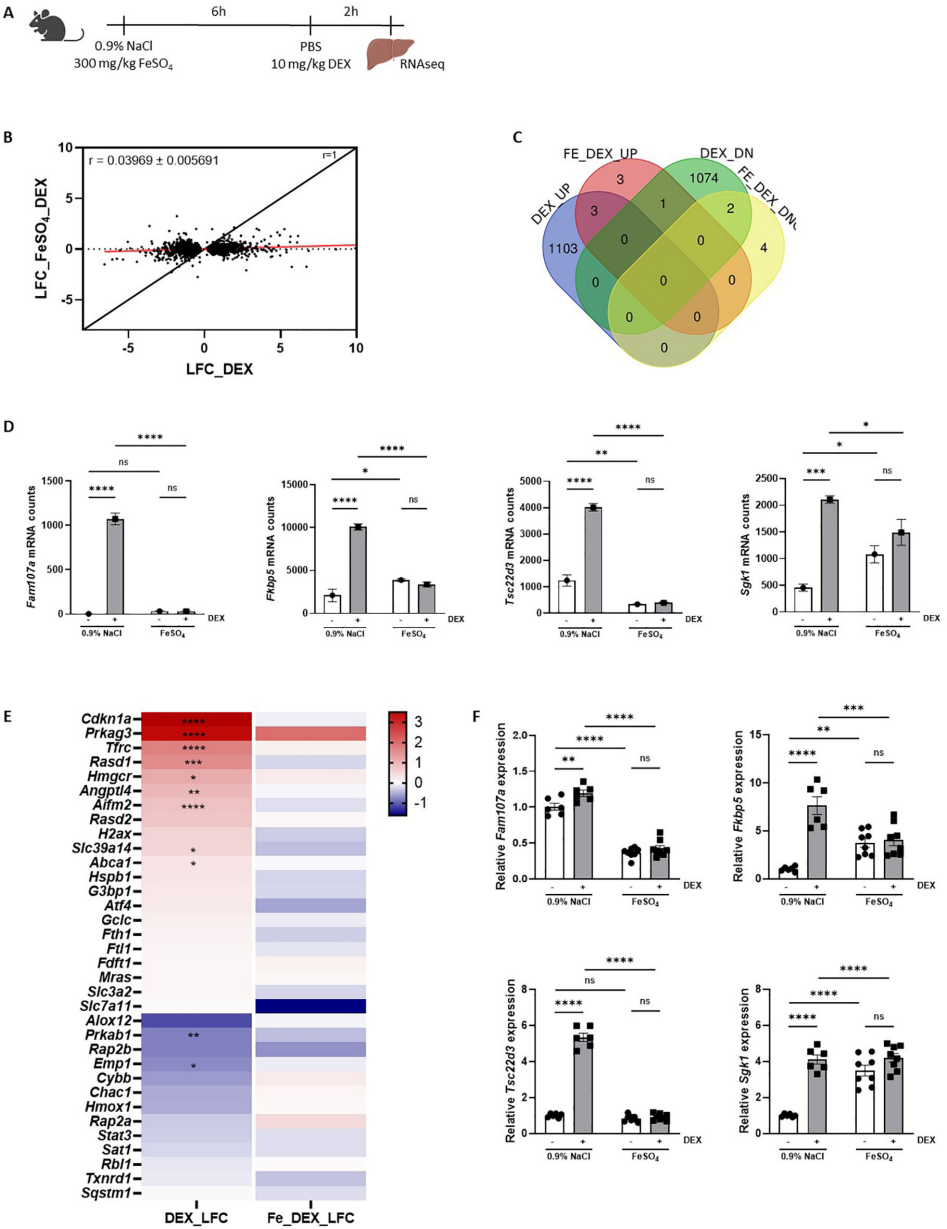
FeSO_4_ induces genome-wide GC resistance. **(A)** Experimental setup: C57BL/6J mice were injected with 300 mg/kg FeSO_4_ or 0.9% NaCl, followed by 10 mg/kg DEX stimulation after 6h. Liver was isolated 2h later for RNAseq analysis (n = 3 per group). **(B)** Scatter plot showing the log fold change (LFC) of all DEX-upregulated genes (FDR < 0.05, LFC > 0) and DEX-downregulated genes (FDR < 0.05, LFC < 0) in 0.9% NaCl versus 300 mg/kg FeSO_4_ mice after 6h. The black line represents the diagonal (r = 1), and the red line represents the real slope ± standard error of the data (r = 0.03969 ± 0.005691) as analyzed by linear regression. **(C)** Venn diagram depicting the number of genes upregulated (UP, FDR < 0.05 and LFC > 0) or downregulated (DOWN, FDR < 0.05 and LFC < 0) by DEX in 0.9% NaCl and FeSO_4_ injected mice. **(D)** Examples of typical GR-responsive genes, the mRNA counts shown are based on the RNAseq data after 0.9% NaCl or FeSO_4_ injection and DEX stimulation. **(E)** Heatmap represents the log_10_ values of the differentially expressed genes (counts) in 300 mg/kg FeSO_4_ injected mice after DEX stimulation which are involved in the ferroptosis signaling pathway identified using IPA. **(F)** C57BL/6J mice were injected with 300 mg/kg FeSO_4_ or 0.9% NaCl, followed by 10 mg/kg DEX stimulation after 6h. Kidney was isolated 2h later. The expression of typical GR-responsive genes was measured via RT-qPCR. N = 6-8 per group, two independent experiments. Data information: All bars represent mean ± SEM. P-values were calculated using two-way ANOVA followed by *post-hoc* Šídák’s multiple comparisons test to correct for multiple testing during the pairwise multiple comparisons, except if otherwise stated. ****P ≤ 0.0001; ***P ≤ 0.001; **P ≤ 0.01; *P ≤ 0.05; ns, not significant.

Our findings demonstrate that endogenous GC/GR signaling is crucial for the tolerance of an acute iron overload but that the therapeutic efficacy of exogenous GC treatment appears to be absent. This dilemma may be attributed to GC resistance present in the liver, as we have shown to occur in polymicrobial sepsis (16) and/or in other organs. To assess whether GC resistance is induced by FeSO_4_, GR activity was studied on a genome-wide scale during ferroptosis progression. Mice were injected i.p. with either 0.9% NaCl or FeSO_4_ (300 mg/kg), followed by an i.p. injection of PBS or 10 mg/kg DEX after 6h to stimulate GR transcriptional activity. The livers were isolated from these mice 2h after PBS/DEX injection and bulk RNAseq was performed (**Figure 4A**).

When plotting the LFC of all DEX responsive genes (|LFC| > 0 and FDR ≤ 0.05) following FeSO_4_ and 0.9% NaCl injection, a significant reduction in GR response to DEX was observed in FeSO_4_-injected mice (**Figure 4B**, slope ± standard error of linear regression curve (LRC) = 0.03969 ± 0.007096), indicating that a clear lack of GR response to DEX (GC resistance) is present in the liver. In total, 1.106 genes were significantly upregulated by DEX in the control group (LFC > 1, FDR ≤ 0.05), while only 7 genes were upregulated by DEX in the presence of FeSO_4_. Similarly, 1.077 genes were downregulated by DEX (LFC < -1, FDR ≤ 0.05) in the 0.9% NaCl condition, compared to only 6 genes in the FeSO_4_ group (**Figure 4C**). Typical GR-responsive genes are displayed in **Figure 4D**. Furthermore, the expression level of genes involved in ferroptosis signaling identified *via* IPA (**Figure 1D**) were clearly induced by DEX in the control group while their expression levels were no longer altered in the presence of FeSO_4_ (**Figure 4E**). As the kidney also plays an important role in iron homeostasis alongside the liver and therefore could be affected by FeSO_4_ administration (46), the GR response in the kidneys was studied using qPCR. **Figure 4F** shows that DEX-induced gene expression occurred in the kidney in the 0.9% NaCl group, but not in the FeSO_4_-treated group. Taken together, acute iron overload induces a very severe form of GC resistance in both the liver and the kidney, significantly impairing the response to exogenous or endogenous GCs. Based on the data shown in **Figure 1**, it thus appears that FeSO_4_ first activates the HPA axis, leading to some GR stimulation in the liver, but that the GR is quickly attacked by Fe, completely whipping out its activity.

### 3.5 GC resistance during FeSO_4_-induced lethal shock is a consequence of a reduced nuclear translocation of GR

**Figure 5:**
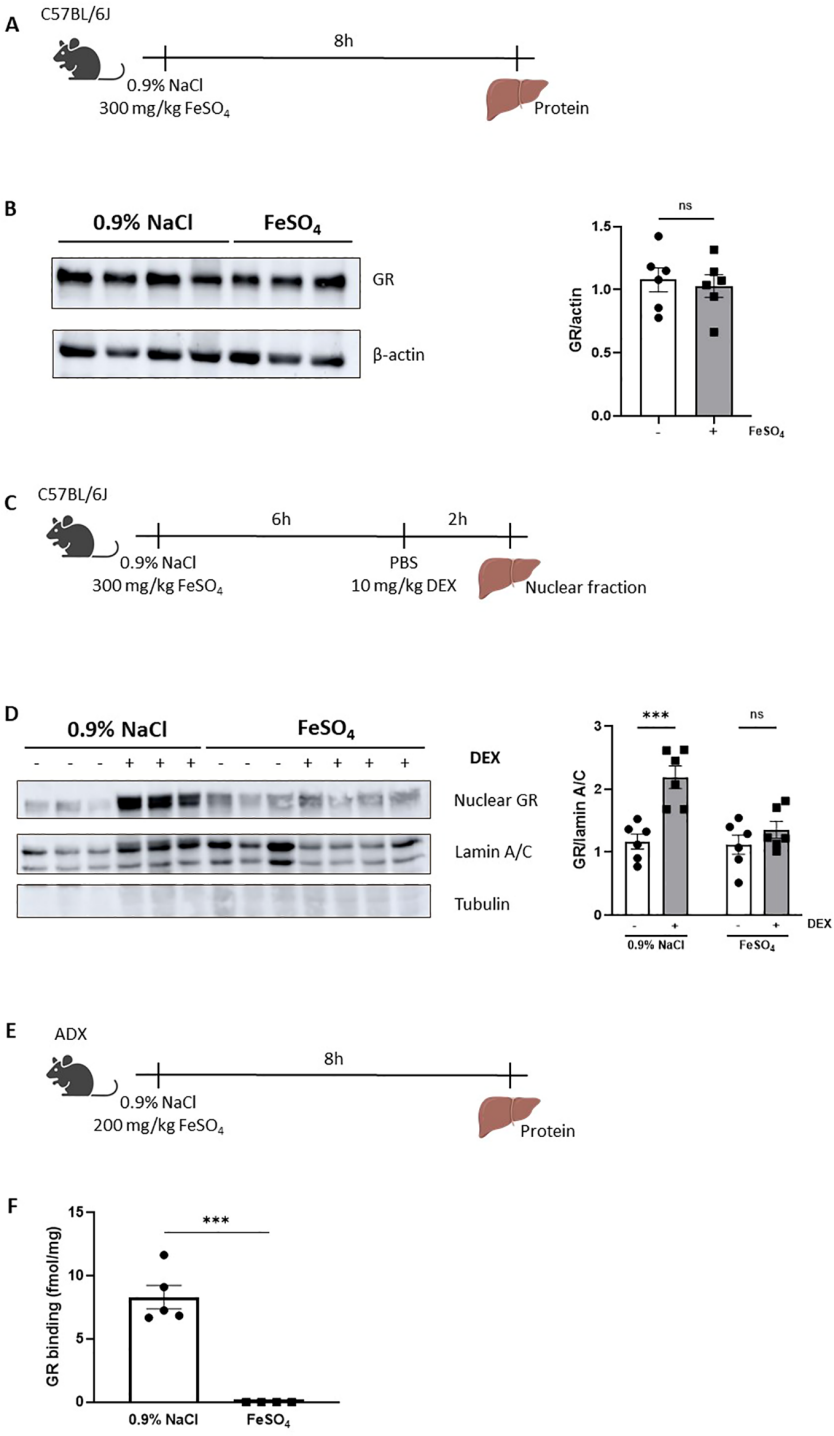
GC resistance during FeSO_4_-induced lethal shock is a consequence of a reduced nuclear translocation of GR. **(A)** Experimental setup: C57BL/6J mice were injected with 300 mg/kg FeSO_4_ or 0.9% NaCl, and livers were isolated after 8h for protein isolation. **(B)** GR protein levels (94 kDa) in the liver were analyzed *via* western blot using β-actin (42 kDa) as a loading control. GR protein levels were visualized and quantified using Amersham Imager 600. N = 2–4 per group, two independent experiments. Representatives immunoblot is depicted. Each dot represents a single biological replicate. P-values were calculated using Mann-Whitney test. **(C)** Experimental setup for liver nuclear fraction extraction: C57BL/6J mice were injected with 300 mg/kg FeSO_4_ or 0.9% NaCl, followed by 10 mg/kg DEX stimulation after 6h. Liver nuclear fractions were isolated 2h later. **(D)** Western blot analysis of GR protein levels in nuclear extract of mouse livers. GR bands (94 kDa) were normalized to the intensities of Lamin A/C bands (74/65 kDa), a marker for nuclear extract. β-tubulin (50 kDa) was used as a control for cytoplasmic contamination. GR protein levels were visualized and quantified using Amersham Imager 600. N = 2–4 per group, two independent experiments. Representative immunoblot is depicted. Each dot represents a single biological replicate. **(E)** Experimental setup for liver cytosol preparation: ADX mice were injected with 200 mg/kg FeSO_4_ or 0.9% NaCl, and livers were isolated after 8h. **(F)** GR protein-specific binding capacity in liver. N = 4-5 per group. P-values were calculated using Mann-Whitney test. Data information: All bars represent mean ± SEM. P-values were calculated using two-way ANOVA followed by *post-hoc* Šídák’s multiple comparisons test to correct for multiple testing during the pairwise multiple comparisons, except if otherwise stated. ***P ≤ 0.001; ns, not significant.

To investigate the potential mechanisms of GC resistance during acute iron overload, several key aspects of the GR signaling pathway were examined after injection either 0.9% NaCl or 300 mg/kg FeSO_4_ followed by DEX stimulation. The GC signaling pathway includes several critical processes, such as binding of GCs to cytoplasmic GR, nuclear translocation of the receptor, DNA binding, and subsequent regulation of gene expression (47). First, GR protein levels were measured in livers of mice isolated 8h after FeSO_4_ injection using western blot (**Figure 5A**). No significant differences in GR protein levels were observed between the 0.9% NaCl and FeSO_4_ groups (**Figure 5B**). Next, we investigated the nuclear translocation of GR after DEX stimulation. Mice were injected i.p. with 0.9% NaCl or FeSO_4_ (300 mg/kg) and 6h later stimulated with DEX or PBS. The liver was isolated after 2h and nuclear fractions were isolated as shown in **Figure 5C**. Western blot analysis confirmed that DEX treatment induced nuclear import of GR in the 0.9% NaCl group, however this translocation was significantly impaired under acute iron overload conditions (**Figure 5D**). Furthermore, GR levels in the cytoplasm decreased significantly in the 0.9% NaCl group, while no changes were observed in the FeSO_4_ group (**Supplementary Figure 4A**). Collectively, these findings demonstrate that GR nuclear translocation is disrupted by an acute iron overload.

Before GR undergoes nuclear translocation, ligand binding (endogenous GCs or synthetic GCs such as DEX) occurs in the cytoplasm. This ligand-binding event induces a conformational change of the GR, resulting in its dissociation from the molecular chaperone complex, exposing the NLS which enables the recruitment of the nuclear import machinery and facilitating the translocation of the GC-GR complex into the nucleus. To understand why the nuclear translocation of GR is disrupted, we investigated whether the ligand binding capacity of the GR might be affected after acute iron overload. To eliminate the potential effects of endogenous GCs, ADX mice were used. Given the high sensitivity of ADX mice to iron, the FeSO_4_ dose was adjusted to the maximum tolerated dose for an 8h period. Liver tissue was isolated 8h after injection of 0.9% NaCl or 200 mg/kg FeSO_4_ (**Figure 5E**). Liver cytosol was prepared, and GR binding capacity was assessed using radiolabeled corticosterone. We found that FeSO_4_ significantly reduced the ability of GR to bind corticosterone (**Figure 5F**). In conclusion, GC resistance observed during acute iron overload is attributed to the disruptive effects of iron on the GR ligand binding capacity, ultimately impairing its nuclear translocation and transcriptional function.

## 4 Discussion

Iron is an essential element required for various physiological processes, including oxygen transport, DNA synthesis, and energy metabolism. However, excessive iron is toxic and is implicated in the pathogenesis of various diseases (1, 2). Recent studies provide compelling evidence that elevated plasma iron levels are associated with poor outcomes in critically ill patients in intensive care units (ICUs) (5). In sepsis in particular, the severity of tissue damage and multiple organ dysfunction syndrome (MODS) is directly correlated with the extent of iron accumulation (25). Van Coillie et al. introduced an acute iron overload mouse model to induce MODS and found that excessive iron significantly activates the ferroptosis pathway, leading to iron accumulation and lipid peroxidation (26). Since the liver has important synthetic, storing, and regulatory functions in iron homeostasis (48), and GC/GR signaling is involved in several metabolic processes in the liver such as glucose metabolism, fatty acid metabolism, and bile acid metabolism (49), we wanted to investigate the crosstalk between iron and GC/GR biology in more detail with the liver as a central hub. As the primary iron storage organ, the liver is especially vulnerable to iron-induced toxicity (33, 50). To further explore this, we employed this acute iron overload model and conducted RNAseq analyses on the liver.

Our results reveal that the ferroptosis signaling pathway was activated by acute iron overload. This finding aligns with previous studies (26). In addition to ferroptosis, iron also triggers inflammatory responses. Jisen et al. have shown that iron increases levels of the pro-inflammatory cytokine IL-6 in a dose-dependent manner in mouse epithelial JB6 cells (51). Similarly, Yuxiao et al. reported that dextran-iron injection elevated hepatic IL-6 mRNA expression in mice (52). Van Coillie et al. also observed increased IL-6 levels in plasma following acute FeSO_4_ injection in mice (26). Consistently, our IPA pathway analysis suggests that FeSO_4_ activates the IL-6 signaling pathway, contributing to inflammation, which was confirmed *via* serum IL-6 levels. Importantly, we also observed the activation of hepatic GR signaling under acute iron overload. This activation appeared to be dependent on HPA axis stimulation rather than direct activation by iron itself. This finding is supported by prior studies demonstrating that iron overload increases the levels of stress-related metabolites, such as corticosterone (22). Together, acute iron overload activates ferroptosis and inflammatory signaling pathways in the liver, while also stimulating a systemic stress response dependent on the HPA axis causing quick GR activation.

The anti-inflammatory properties of the GC/GR signaling pathway are well-documented, with GR activation suppressing excessive inflammation by inhibiting the production of pro-inflammatory cytokines, such as IL-1β, IL-6, and IFN-β (53). Moreover, the GR pathway plays a role in regulating iron homeostasis through the modulation of genes involved in iron transport and storage in liver (23). Considering the potential for GC signaling to mitigate iron-induced lethality, we hypothesized that GR activation might counteract the harmful effects of iron overload, providing a feedback mechanism to protect against excessive iron-induced inflammation, ferroptosis and tissue damage. However, most of the current research is focusing on the effect of GC/GR in iron metabolism under normal physiological conditions, rather than iron overload models. For instance, He et al. found that GCs promote iron accumulation in the liver by upregulating the expression of IRP-1 (23). Similarly, corticosterone exposure in hippocampal neurons has been shown to increase intracellular iron levels while decreasing ferritin expression (24). Conversely, other studies suggest that long-term DEX exposure reduces liver iron content, which correlates with the downregulation of hepatic TFR1 protein expression (54).

Our study addresses the role of GR signaling during iron overload and reveals that blocking the GC/GR pathway in mice results in extreme sensitivity to most iron-induced toxic effects, including lethality. Even low doses of iron in this context led to lethality, accompanied by significant iron accumulation in both the serum and liver, elevated serum MDA levels, and tissue injury markers. These data suggest a critical role of GR signaling in maintaining iron homeostasis during iron overload conditions with an essential role for GR in resisting the toxic effects of iron overload. Moreover, we observed that inhibition of GR signaling under iron overload conditions led to the upregulation of genes involved in iron uptake (*Tfrc* and *Slc39a14*) and storage (*Fth1*), while expression of the iron export gene ferroportin (*Fpn*) was reduced. This suggests that GR activation normally serves to limit iron accumulation and mitigate oxidative stress, providing a protective mechanism during iron excess. In contrast to these data, we observed that stimulating GR by the common synthetic ligand DEX had no protective effect. Such a situation, in which GR is essential to protect, but cannot be stimulated in a therapeutic way, was shown in polymicrobial sepsis, which appeared to result from a profound inactivity of GR, called GC resistance (16).

Indeed, we observed a significant GC resistance in both the liver and kidney under iron overload conditions. GC resistance is characterized by reduced sensitivity or responsiveness to GCs, despite normal or elevated levels of these hormones (19). Our findings provide a mechanistic explanation for the failure of protection by DEX in the acute iron overload model. Understanding the mechanisms underlying GC resistance is crucial for developing targeted therapeutic strategies. The impaired GC response in GC resistance may arise from various mechanisms, including alterations in the GR, dysregulation of GR signaling pathways, increased expression of pro-inflammatory cytokines, elevated levels of the dominant-negative GRβ isoform, or post-translational modifications affecting GR function (19). In sepsis for instance, studies have demonstrated that GR-DNA binding is significantly diminished in CLP-induced sepsis models during the early stages of disease progression, underscoring a disruption in GR signaling in this condition (16). Here, our findings suggest a distinct mechanism in acute iron overload, where iron directly impairs the GR-ligand binding affinity. To eliminate the potential influence of endogenous GCs, we utilized ADX mice (55) to assess GR binding affinity thereby focusing on the direct effects of iron overload on GR-ligand interactions without the confounding impact of endogenous GC production. Iron overload prevented the proper nuclear translocation of the GR-ligand complex and inhibits subsequent transcriptional activation of GR-responsive genes. Unlike sepsis, acute iron overload specifically alters GR function at the ligand-binding level. The exact molecular mechanisms by which iron interferes with GR-ligand interactions remain unclear and warrants further investigation. One attractive possibility is that, like has been shown for the estrogen receptor under certain conditions, the Zn^2+^ ions that coordinate the cysteine residues in both GR Zn fingers, one of which is essential for GR dimerization and the other one for DNA binding, in the presence of Fe^2+^ overload, might be exchanged to Fe-fingers rather than Zn fingers (56). However, this point remains speculation, and it would also be hard to explain why this mechanism leads to a lack of GR ligand binding.

Taken together, our results provide strong evidence that acute iron overload induces GC resistance by impairing GR ligand-binding capacity. This impairment likely plays a crucial role in the pathophysiology of iron overload-associated disorders, potentially contributing to the dysregulation of inflammatory responses and exacerbating disease progression in conditions of iron excess.

## Data Availability

The datasets presented in this study can be found in online repositories. The names of the repository/repositories and accession number(s) can be found below: https://www.ncbi.nlm.nih.gov/geo/, GSE290955.

## Supplementary material

Supplementary Figure 1
**(A)** C57BL/6J mice were injected i.p. with different dose of FeSO_4_ (200 mg/kg, 250 mg/kg, 275 mg/kg, 300 mg/kg). Survival was monitored. N-values are indicated in the legend. **(B)** C57BL/6J mice were injected i.p. with 300 mg/kg of Na_2_SO_4_, 300 mg/kg of NaCl, and 300 mg/kg of FeSO_4_. Survival was monitored. N-values are indicated in the legend. **(C)** C57BL/6J mice were injected with 300 mg/kg FeSO_4_ or 0.9% NaCl, and serum was collected 8h after injection. Serum IL-6 levels were measured. N = 6, two independent experiments. P-values were calculated using Mann-Whitney test. **(D)** ADX or C57BL/6J mice were injected with 300 mg/kg FeSO_4_ or 0.9% NaCl, and liver RNA was isolated 2h after injection. The expression of typical GR-responsive genes was measured *via* RT-qPCR. N = 3-7, one experiment. Data information: All bars represent mean ± SEM. P-values were calculated using two-way ANOVA followed by *post-hoc* Šídák’s multiple comparisons test to correct for multiple testing during the pairwise multiple comparisons, except if otherwise stated. Survival curves were analyzed with a Log-Rank (Mantel-Cox) test. ****P ≤ 0.0001; ***P ≤ 0.001; *P ≤ 0.05; ns, not significant.

Supplementary Figure 2
**(A, B)** C57BL/6J mice were pretreated for 30 minutes with 10 mg/kg DEX or PBS, followed by injection of 0.9% NaCl or 300 mg/kg FeSO_4_. Serum and liver were collected 8h later for analysis. Fe³^+^ and total iron concentrations in serum **(A)** and liver **(B)** were measured. N = 6, two independent experiments. Data information: All bars represent mean ± SEM. P-values were calculated using two-way ANOVA followed by *post-hoc* Šídák’s multiple comparisons test to correct for multiple testing during the pairwise multiple comparisons, except if otherwise stated. ****P ≤ 0.0001; ***P ≤ 0.001; **P ≤ 0.01; *P ≤ 0.05; ns, not significant.

Supplementary Figure 3
**(A, B)** C57BL/6J mice were pretreated for 30 minutes with 5 mg RU486 or vehicle (DMSO), followed by an injection of 0.9% NaCl or 150 mg/kg FeSO_4_. Serum and liver were collected 8h later for analysis. **(A)** Fe^3+^ and total iron concentrations in serum and liver were measured. N=6, two independent experiments. **(B)** The expressions of typical genes involved in iron metabolism were measured *via* RT-qPCR in the liver. Data information: All bars represent mean ± SEM. P-values were calculated using two-way ANOVA followed by *post-hoc* Šídák’s multiple comparisons test to correct for multiple testing during the pairwise multiple comparisons, except if otherwise stated. ****P ≤ 0.0001; ***P ≤ 0.001; **P ≤ 0.01; *P ≤ 0.05; ns, not significant.

Supplementary Figure 4
**(A)** C57BL/6J mice were injected with 300 mg/kg FeSO_4_ or 0.9% NaCl, followed by 10 mg/kg DEX stimulation after 6h. Liver cytosolic fractions were isolated 2h later. Western blot analysis of GR protein levels in cytosolic extract of mouse livers. GR bands (94 kDa) were normalized to the intensities of β-tubulin (50 kDa) bands. GR protein levels were visualized and quantified using Amersham Imager 600. N = 6 per group, two independent experiments. Representative immunoblot is depicted. Each dot represents a single biological replicate. All bars represent mean ± SEM. P-values were calculated using two-way ANOVA followed by post-hoc Šídák’s multiple comparisons test to correct for multiple testing during the pairwise multiple comparisons. *P ≤ 0.05; ns, not significant.
