# Supplementary figures and images for: Modulation of glucocorticoid receptor function under iron overload
Source: Front Immunol. 2025 Jun 18;16:1605420. doi: 10.3389/fimmu.2025.1605420 (PMC12213461; doi:10.3389/fimmu.2025.1605420)

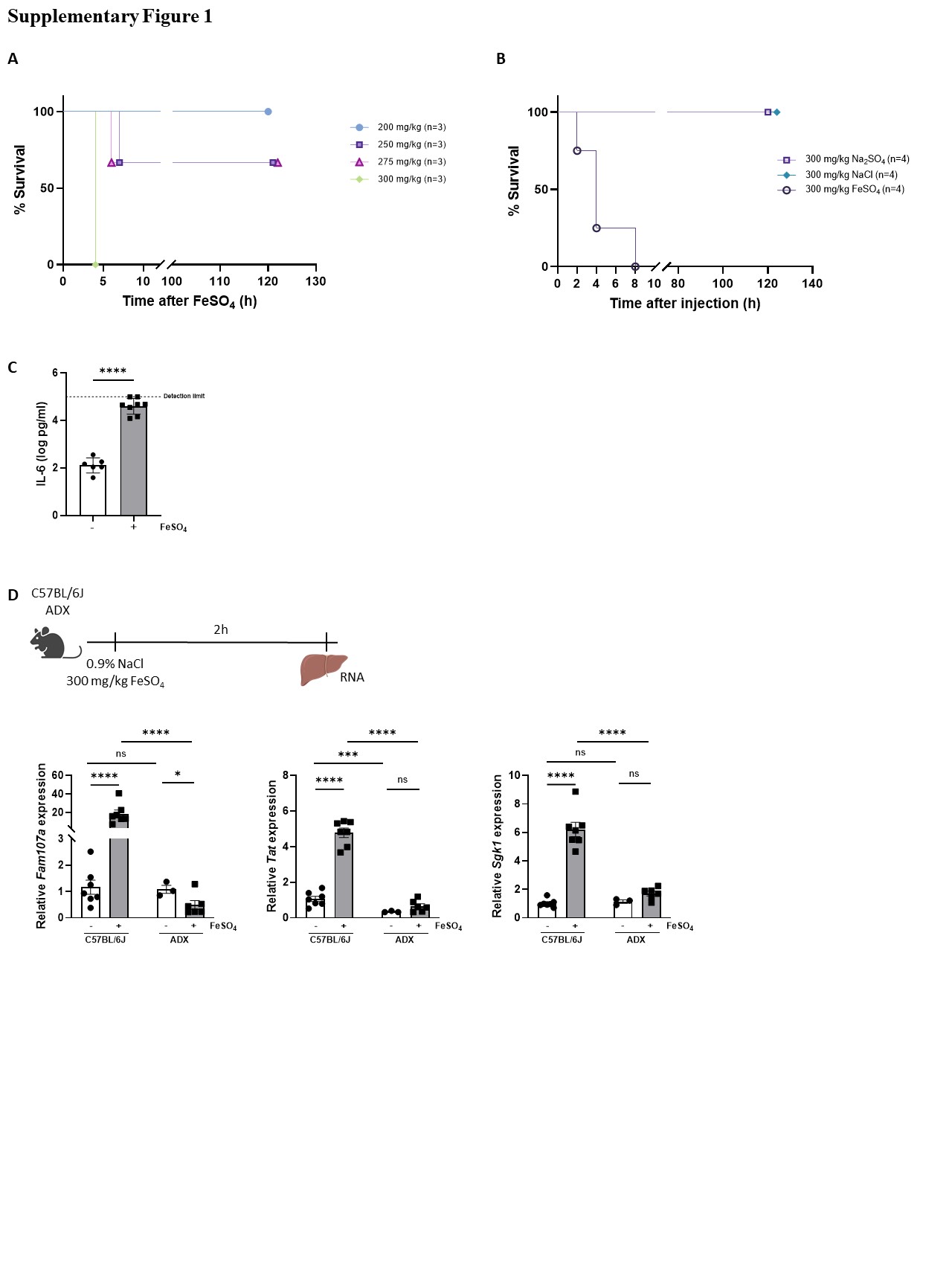

Supplement: Supplementary Figure 1 — (A) C57BL/6J mice were injected i.p. with different dose of FeSO4 (200 mg/kg, 250 mg/kg, 275 mg/kg, 300 mg/kg). Survival was monitored. N-values are indicated in the legend. (B) C57BL/6J mice were injected i.p. with 300 mg/kg of Na2SO4, 300 mg/kg of NaCl, and 300 mg/kg of FeSO4. Survival was monitored. N-values are indicated in the legend. (C) C57BL/6J mice were injected with 300 mg/kg FeSO4 or 0.9% NaCl, and serum was collected 8h after injection. Serum IL-6 levels were measured. N = 6, two independent experiments. P-values were calculated using Mann-Whitney test. (D) ADX or C57BL/6J mice were injected with 300 mg/kg FeSO4 or 0.9% NaCl, and liver RNA was isolated 2h after injection. The expression of typical GR-responsive genes was measured via RT-qPCR. N = 3-7, one experiment. Data information: All bars represent mean ± SEM. P-values were calculated using two-way ANOVA followed by post-hoc Šídák’s multiple comparisons test to correct for multiple testing during the pairwise multiple comparisons, except if otherwise stated. Survival curves were analyzed with a Log-Rank (Mantel-Cox) test. ****P ≤ 0.0001; ***P ≤ 0.001; *P ≤ 0.05; ns, not significant. [file Image1.jpg]

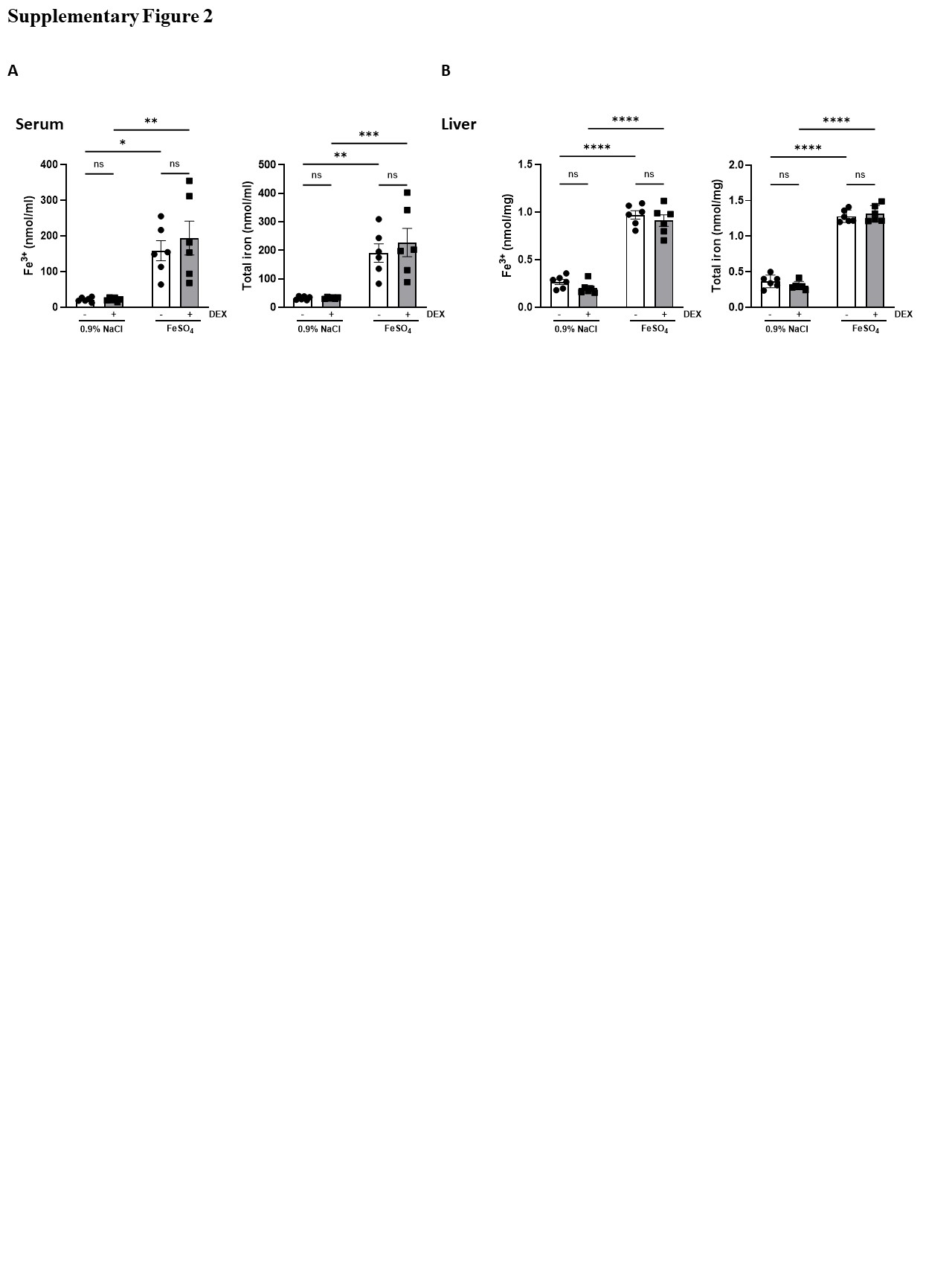

Supplement: Supplementary Figure 2 — (A, B) C57BL/6J mice were pretreated for 30 minutes with 10 mg/kg DEX or PBS, followed by injection of 0.9% NaCl or 300 mg/kg FeSO4. Serum and liver were collected 8h later for analysis. Fe³+ and total iron concentrations in serum (A) and liver (B) were measured. N = 6, two independent experiments. Data information: All bars represent mean ± SEM. P-values were calculated using two-way ANOVA followed by post-hoc Šídák’s multiple comparisons test to correct for multiple testing during the pairwise multiple comparisons, except if otherwise stated. ****P ≤ 0.0001; ***P ≤ 0.001; **P ≤ 0.01; *P ≤ 0.05; ns, not significant. [file Image2.jpg]

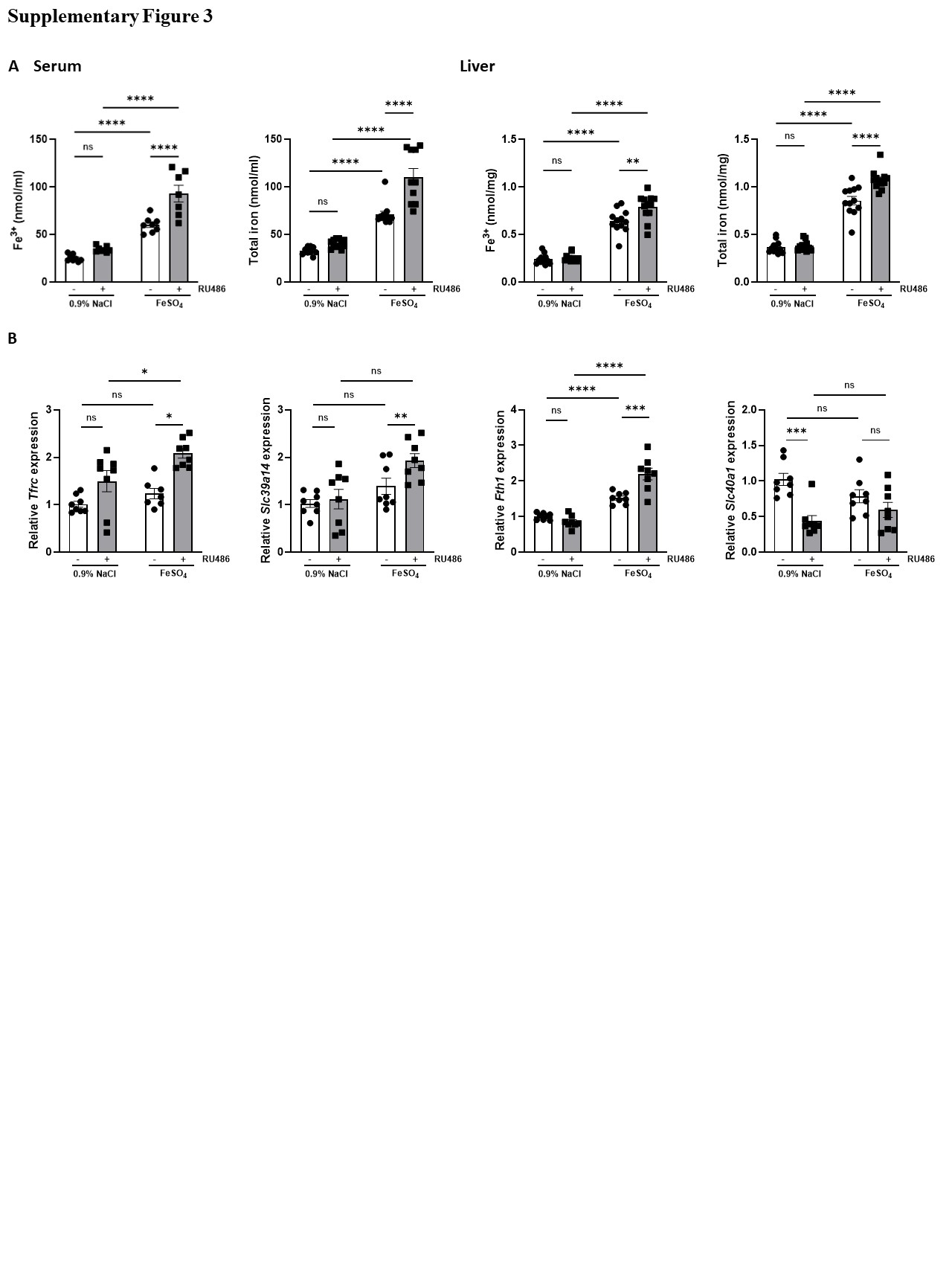

Supplement: Supplementary Figure 3 — (A, B) C57BL/6J mice were pretreated for 30 minutes with 5 mg RU486 or vehicle (DMSO), followed by an injection of 0.9% NaCl or 150 mg/kg FeSO4. Serum and liver were collected 8h later for analysis. (A) Fe3+ and total iron concentrations in serum and liver were measured. N=6, two independent experiments. (B) The expressions of typical genes involved in iron metabolism were measured via RT-qPCR in the liver. Data information: All bars represent mean ± SEM. P-values were calculated using two-way ANOVA followed by post-hoc Šídák’s multiple comparisons test to correct for multiple testing during the pairwise multiple comparisons, except if otherwise stated. ****P ≤ 0.0001; ***P ≤ 0.001; **P ≤ 0.01; *P ≤ 0.05; ns, not significant. [file Image3.jpg]

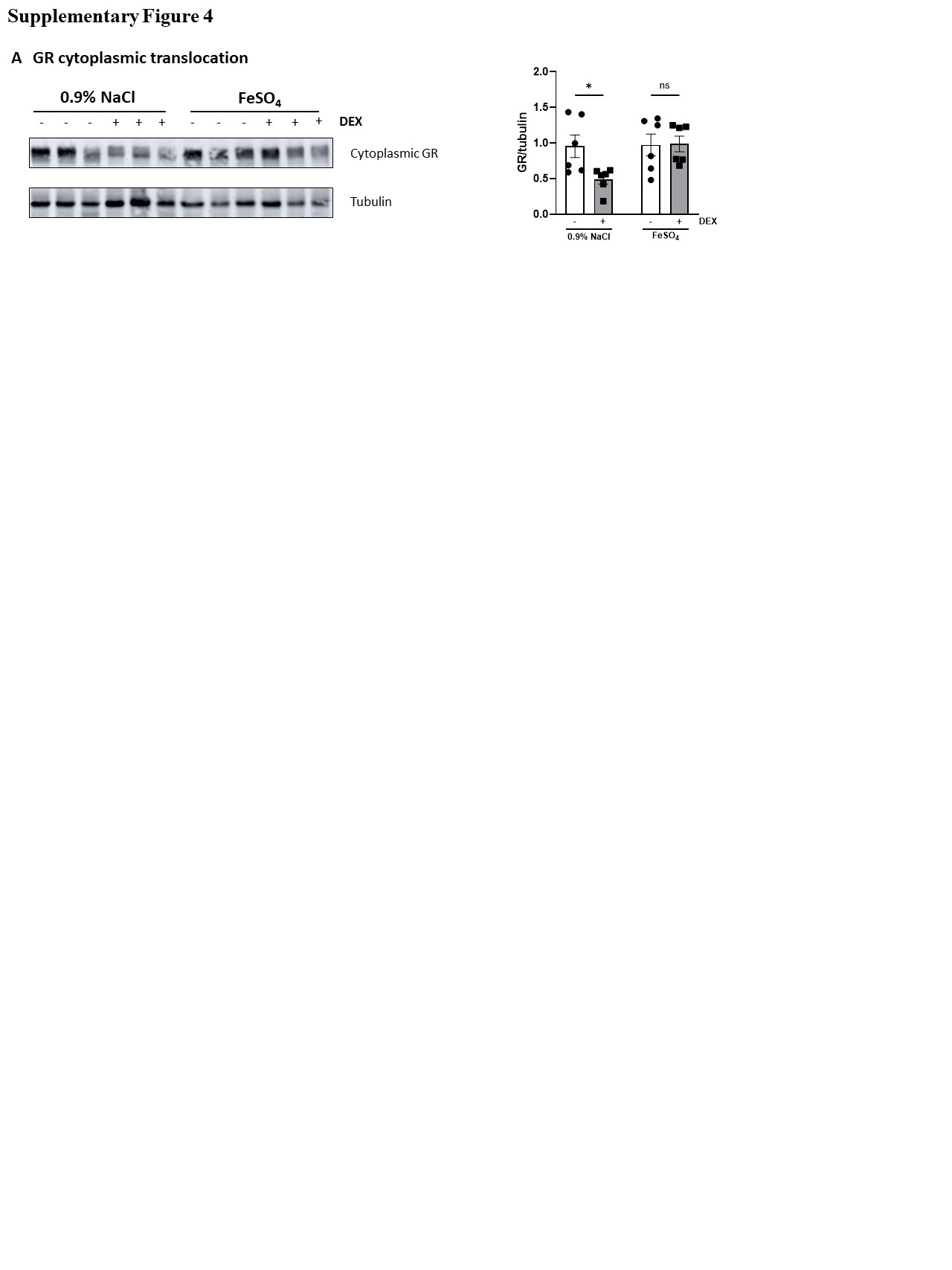

Supplement: Supplementary Figure 4 — (A) C57BL/6J mice were injected with 300 mg/kg FeSO4 or 0.9% NaCl, followed by 10 mg/kg DEX stimulation after 6h. Liver cytosolic fractions were isolated 2h later. Western blot analysis of GR protein levels in cytosolic extract of mouse livers. GR bands (94 kDa) were normalized to the intensities of β-tubulin (50 kDa) bands. GR protein levels were visualized and quantified using Amersham Imager 600. N = 6 per group, two independent experiments. Representative immunoblot is depicted. Each dot represents a single biological replicate. All bars represent mean ± SEM. P-values were calculated using two-way ANOVA followed by post-hoc Šídák’s multiple comparisons test to correct for multiple testing during the pairwise multiple comparisons. *P ≤ 0.05; ns, not significant. [file Image4.jpg]
